# Melatonin Secretion during a Short Nap Fosters Subsequent Feedback Learning

**DOI:** 10.3389/fnhum.2017.00648

**Published:** 2018-01-10

**Authors:** Christian D. Wiesner, Valentia Davoli, David Schürger, Alexander Prehn-Kristensen, Lioba Baving

**Affiliations:** ^1^Department of Clinical Psychology and Psychotherapy, Institute of Psychology, Christian-Albrechts-University zu Kiel, Kiel, Germany; ^2^Department of Child and Adolescent Psychiatry and Psychotherapy, School of Medicine, Christian-Albrechts-University zu Kiel, Kiel, Germany

**Keywords:** sleep, melatonin, reward, dopaminergic system, striatum-dependent, probabilistic learning, feedback learning, working memory

## Abstract

Sleep helps to protect and renew hippocampus-dependent declarative learning. Less is known about forms of learning that mainly engage the dopaminergic reward system. Animal studies showed that exogenous melatonin modulates the responses of the dopaminergic reward system and acts as a neuroprotectant promoting memory. In humans, melatonin is mainly secreted in darkness during evening hours supporting sleep. In this study, we investigate the effects of a short period of daytime sleep (nap) and endogenous melatonin on reward learning. Twenty-seven healthy, adult students took part in an experiment, either taking a 90-min afternoon nap or watching videos (within-subject design). Before and after the sleep vs. wake interval, saliva melatonin levels and reward learning were measured, and in the nap condition, a polysomnogram was obtained. Reward learning was assessed using a two-alternative probabilistic reinforcement-learning task. Sleep itself and subjective arousal or valence had no significant effects on reward learning. However, this study showed for the first time that an afternoon nap can elicit a small but significant melatonin response in about 41% of the participants and that the magnitude of the melatonin response predicts subsequent reward learning. Only in melatonin responders did a short nap improve reward learning. The difference between melatonin-responders and non-responders occurred very early during learning indicating that melatonin might have improved working memory rather than reward learning. Future studies should use paradigms differentiating working memory and reward learning to clarify which aspect of human feedback learning might profit from melatonin.

## Introduction

The ability to learn from experiences and thereby adapt behavior to opportunities and challenges is vital to animals living in a changing environment (Dunlap and Stephens, 2009). Reward learning is one such essential form of learning which enables animals to learn to prefer actions or choice alternatives that are frequently followed by reward (Schultz, 2015). Here we look at possible mechanisms that renew this valuable asset of reward-learning ability in humans. To this end, we focus on the effects of sleep and melatonin on subsequent reward learning.

In the past decade, numerous studies have shown that *sleep* can foster subsequent learning (Yoo et al., 2007; Van Der Werf et al., 2009, 2011; Mander et al., 2011; Antonenko et al., 2013; Kaida et al., 2015). Nevertheless, it is still unclear which forms of learning profit from previous sleep. For example, Van Der Werf et al. (2009) found that a mild disruption of sleep during the night impaired post-sleep declarative encoding of pictures. In a nap study, Mander et al. (2011) showed that the episodic encoding capacity deteriorated during the day and was restored by a short nap. However, motor skill learning was not affected by wake or sleep at all. The same workgroup also reported that total sleep deprivation resulted in deficient hippocampal activity during encoding and worse episodic encoding performance (Yoo et al., 2007). This matches the results from two studies using overnight, total sleep deprivation, or selective REM-sleep deprivation (Kaida et al., 2015). The authors found that total sleep deprivation impaired subsequent declarative encoding of pictures. However, REM-sleep deprivation did not affect subsequent declarative encoding. In another study by the same workgroup, enhancing slow-wave activity by transcranial slow oscillation stimulation increased subsequent encoding capacity in several declarative tasks but not in a procedural finger-tapping task (Antonenko et al., 2013). In summary, there is solid evidence that even short periods of sleep renew hippocampus-dependent, declarative learning capacity. In contrast, procedural learning, especially motor skill learning, does not seem to profit from previous sleep. More importantly, there is a lack of studies investigating whether sleep also fosters subsequent reward learning. Reward learning and motor skill learning are both procedural and in part rely on similar frontostriatal circuits (Alexander et al., 1986; Hayes et al., 2015; Schultz, 2015). Therefore, from an empirical standpoint, one might suspect that sleep does not foster subsequent reward learning either.

From a theoretical standpoint, one might still expect that slow-wave sleep or REM sleep do foster subsequent reward learning. For example, the synaptic homeostasis hypothesis points out that learning during wakefulness results in a net increase in synaptic strength in the whole brain which is supposed to decrease signal-to-noise ratios and saturate learning (Tononi and Cirelli, 2014). According to this hypothesis, sleep, especially slow-wave sleep, is supposed to renormalize synaptic weights thereby refreshing the ability to learn night by night. Other authors highlight the role of REM-sleep in reward processing (Perogamvros and Schwartz, 2012, 2015). Indeed, some studies recording spike activity of dopaminergic neurons in the ventral tegmental area of the rat have shown that this part of the “reward system” is highly active during paradoxical sleep (Dahan et al., 2007; Valdes et al., 2015). In line with this, REM-sleep deprivation has been shown to impair subsequent operant conditioning in rats (Hanlon et al., 2005). Less is known about the influence of REM sleep on reward learning in humans. Although total sleep deprivation seems to impair subsequent reward-related decision-making and feedback learning (Whitney et al., 2015), potentially positive effects of slow-wave sleep or REM sleep on reward learning remain elusive. Therefore, we investigate whether an afternoon nap can enhance subsequent reward learning. Referring to the animal studies described above, we expect that especially REM sleep will foster subsequent reward learning in humans.

A second mechanism that might foster reward learning is the hormone *melatonin*. The endogenous melatonin secretion depends on the circadian rhythm and in humans usually peaks at night when it is dark (Pandi-Perumal et al., 2007). However, most research on the impact of melatonin on learning ability is done using externally administered instead of endogenously secreted melatonin. In rodents, exogenous melatonin facilitates learning (Zakaria et al., 2016) and modulates the reward system (Clough et al., 2014; Yahyavi-Firouz-Abadi et al., 2007). Furthermore, melatonin, given as a long-term supplement, has been shown to protect the brain against oxidative stress (Miller et al., 2015), neuroinflammation (Hardeland et al., 2015), and other neurodegenerative processes (Polimeni et al., 2014). Of note, melatonin is also known to alleviate the negative effects of sleep-deprivation on hippocampus function (Zhang et al., 2013; Kwon et al., 2015) and memory as well (Alzoubi et al., 2016). However, less is known about the acute effects of externally administered melatonin, let alone endogenous melatonin, on learning ability in humans. To our knowledge, the only study focusing on the impact of acute melatonin on learning in humans was done by Rimmele et al. (2009). Using a single-blind, between-subjects design, the authors showed that 3 mg of melatonin as compared to placebo increased memory acquisition under stress in a hippocampus-dependent declarative memory task. Memory retrieval of words learned during a previous session was not affected by melatonin. Moreover, melatonin did not decrease the secretion of stress hormones like cortisol but seemed to improve memory acquisition independent of cortisol. In summary, it has been shown that exogenous melatonin might improve hippocampus-dependent learning in humans and striatum-dependent reward learning in rodents. However, there is a complete lack of studies investigating the effect of endogenous melatonin on reward learning in humans.

In the present study, we used a within-subject design to investigate whether an afternoon nap as compared to watching a video can improve probabilistic reward learning. Moreover, we conducted a polysomnography and measured endogenous melatonin to shed some light on the question whether sleep or melatonin may foster subsequent reward learning. We expected that REM sleep, as well as melatonin, would improve reward learning.

## Materials and Methods

### Participants

Twenty-seven healthy university students (6 men, 21 women; age: 19–33 years, *M* = 23.6, *SD* = 2.9) participated in the experiment. Three more candidates were recruited but had to be excluded from analysis due to problems falling asleep, inability to produce enough saliva for analysis, or lack of compliance. Potential participants were screened by interview, questionnaires, and protocols. Inclusion criteria were the absence of self-reported history of psychiatric, neurological, or endocrine disorders, a normal amount of somatic and psychiatric symptoms (Symptom-Checklist-90-R; *T*-value of global severity index ≤ 60; Franke, 2002), normal sleep (Pittsburgh Sleep Quality Index; sum-value ≤ 5; Buysse et al., 1989), a regular sleep-wake rhythm (e.g., no shift work, no extreme chronotypes as assessed by interview and sleep-protocols), a body-mass index below 30, and right-handedness (Edinburgh-Handedness Inventory; Oldfield, 1971). Descriptive data are reported in **Table 1**. All participants were free of medication (except hormonal contraceptives in nine women), reported no drug abuse and no nicotine dependence (no habitual smoking and less than five cigarettes per day in 13 participants). The participants were instructed not to smoke or to drink beverages containing caffeine or alcohol for the days of the experiment. Adherence to these instructions and a regular sleep-wake schedule was checked in a debriefing questionnaire. The study was approved by the ethics committee of the Medical Faculty of the University of Kiel. All participants gave written, informed consent prior to participation and were paid 80 Euro at the end of the study.

**Table 1:** Descriptives of questionnaire and sleep data.

Variable	Min	Max	Mean	*SD*
Participant characteristics				
Age (years)	19	33	23.6	2.9
BMI (kg/m^2^)	19.1	24.1	21.9	1.4
SCL-90-R (*T*-value)	27	55	38.1	5.8
PSQI (sum score)	1	5	3.3	1.2
Sleep stages				
S1 (min)	4.5	28.5	13.8	7.0
S2 (min)	2.0	63.0	34.6	14.3
S3 (min)	0.0	12.0	5.4	3.7
S4 (min)	0.0	46.5	9.8	11.7
REM (min)	0.0	16	4.4	5.2
SWS (min)	0.0	50.5	15.2	13.3
TST (min)	7.5	91.0	68.0	21.6
Latency (min)	4.4	62.7	13.8	11.4
Efficiency (%)	8.3	97.5	74.3	23.5

*BMI, body mass index; SCL-90-R, *T*-value of the global severity index of the Symptom-Checklist-90-R; PSQI, sum score of the Pittsburgh-Sleep-Quality Index; S1–S4, sleep stages 1–4 according to Rechtschaffen and Kales (1968); REM, REM-sleep; SWS, slow-wave sleep, i.e., S3 + S4; TST, total sleep time; Latency, sleep-latency to S2; efficiency = 100 ^∗^ TST/time in bed*.

### Probabilistic Learning Task

**FIGURE 1:**
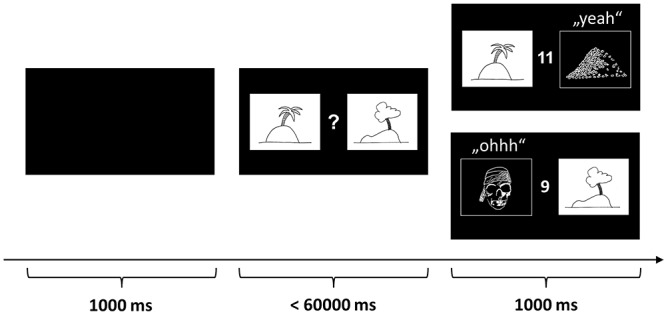
Probabilistic reward-learning task (“pirate game”). The figure depicts one of 33 learning trials in each block. In each trial, two pictures of islands are presented and the participant has to decide which island to explore. If the “correct” island is chosen, the picture of the island is replaced by a picture of a treasure, a sound of children cheering “yeah” is played, and the treasure counter turns green and increases by one (reward). If the “wrong” island is chosen, the island is replaced by a jolly roger, a disappointed voice uttering “ohhh” is played, and the treasure counter turns red and decreases by one (punishment). The participants were instructed to learn by trial and error to approach the island on which a treasure is hidden more often and to avoid the island which is inhabited by pirates more often. The pictures above are merely symbolic. The actual pictures were color photos sampled from the Internet.

To assess the ability to learn from probabilistic feedback, i.e., reward and punishment, we used a task adapted from Pessiglione et al. (2006), which we had used in previous studies with children (Wiesner et al., 2017). In this so-called “pirate game” the participant is asked to explore treasure islands (**Figure 1**). Four equivalent versions of the pirate game with different stimulus sets were programmed in Presentation^®^ software (Version 14.9, Neurobehavioral Systems Inc.) and the versions were approximately pairwise counterbalanced over conditions and order of conditions, e.g., versions A and B before or after the nap or versions C and D before or after the nap. Each version consisted of four blocks of 33 trials and each block used a different pair of pictures. In each trial, two pictures of islands were presented and the participant had to choose which island to explore. Participants indicated their choice by pressing the left or right mouse buttons. If the “correct” island was chosen, the picture of the island was replaced by a picture of a treasure, the sound of children cheering “yeah” was played, and the treasure counter turned green and increased by one (reward). If the “wrong” island was chosen, the island was replaced by a jolly roger, a disappointed voice uttering “ohhh” was played, and the treasure counter turned red and decreased by one (punishment).

During a block of 33 trials, the same islands were repeatedly shown on the left or right side of the monitor in a pseudorandom, counterbalanced order. The participants were instructed to approach the island on which a treasure was hidden more often and to avoid the island which was inhabited by pirates more often. Before and after the manipulations (nap vs. wake) the participants played four blocks of 33 trials. In each block, a unique set of island pictures was used. Probabilistic feedback was provided during each block of 33 trials according to a reinforcement schedule with increasingly valid feedback: In the first third of the trials of each block, the target island was correct with a frequency of 7/11 (≈63.6%) and wrong with a frequency of 4/11 (≈36.4%). In the second third, the reward frequency was increased to 8/11 (≈72.7%) and the punishment frequency decreased to 3/11 (≈27.3%). Finally, in the last third, the reward frequency reached 9/11 (≈81.8%) and the punishment frequency 2/11 (≈18.2%). This schedule was chosen to allow the assessment of a wide range of performance levels. However, a previous study showed that performance differences were only apparent in about the first five trials because the learning curves converged rapidly on the same high level (Wiesner et al., 2017). Therefore, we focused our analysis on the first five trials of each block of trials (also see the section “Data Analysis”).

### Sleep Recording

All participants spent two afternoons in the sleep laboratory. During the training session, the participants were familiarized with the measurement equipment and had the chance to adapt to the conditions while taking a first nap. Furthermore, the data from the adaptation nap were used to exclude severe sleep disorders. The test nap took place at least 1 week later, and sleep was recorded during both naps by standard procedures using a digital electroencephalogram (EEG), electromyogram (EMG), and electrooculogram (EOG). To amplify and record the data, a SOMNOscreen PSG plus (SOMNOmedics, Randersacker, Germany) was used. The EEG was recorded at a sampling rate of 256 Hz with a band-pass filter of 0.4–35 Hz using multi-use Ag/AgCl electrodes attached to the positions C3 and C4 according to the 10–20 system referenced to the contralateral mastoid electrode and with a ground electrode at Fpz. A diagonal EOG was recorded at a sampling rate of 256 Hz with a band-pass filter of 0.2–5 Hz using single-use Ag/AgCl electrodes attached to the lower right and upper left canthi referenced to the contralateral mastoids. Bipolar EMG was recorded at a sampling rate of 256 Hz with a band-pass filter of 10–128 Hz using three single-use Ag/AgCl electrodes attached to the chin (one electrode as a replacement). All sleep data were visually scored according to the criteria by Rechtschaffen and Kales (1968) by a trained rater unaware of the hypotheses. The following macro-sleep parameters were obtained: sleep stages 1–4 and REM sleep (in minutes), time in bed (in minutes), total sleep time (in minutes), sleep-onset latency (time in minutes from lights off to first epoch of sleep stage 2), and sleep efficiency (ratio of total sleep time to time in bed in percent). To control for effects of sleep on mood, the participants rated their mood on the valence and arousal scales of the self-assessment manikin (SAM; Bradley and Lang, 1994) before and after the sleep as well as the wake condition.

### Melatonin Sampling and Analysis

The participants were trained to collect sufficiently large (0.5 mL) and clean saliva samples during the training session. They received written and verbal instructions according to the guidelines published by Pandi-Perumal et al. (2007). During the experimental sessions, samples were collected 45 min and immediately before the manipulation (nap vs. video), immediately after and 45 min after (corresponding to approximately to 2:30, 3:15, 4:45, and 5:30 p.m.). All saliva samples were collected under supervision using cotton swabs (Salivette, Sarstedt, Nümbrecht, Germany), labeled with a code, and then stored at -20°C until the end of data collection. Saliva samples were analyzed using a commercially available competitive, enzyme-linked immunosorbent assay kit (Melatonin direct Saliva ELISA) by the laboratory of the manufacturer (IBL International, Hamburg, Germany). The process has an analytic sensitivity of 0.3 pg/mL, a functional sensitivity of 1 pg/mL, an intra-assay coefficient of variation of 6.1%, and an interassay coefficient of variation of 7.6% in the range of the expected values.

### Design and Procedure

Sleep studies comparing nocturnal sleep vs. daytime wake suffer from confounded circadian processes, namely the increase of melatonin during darkness in the late evening (Pandi-Perumal et al., 2007). Also, nocturnal sleep deprivation is a suboptimal alternative because awakening can be quite stressful and therefore introduce hormonal changes too (von Treuer et al., 1996). Therefore, we decided to employ a nap paradigm, which allowed us to keep the time window in the circadian rhythm constant and avoid stress at the same time.

The levels of the within-subject factor *Condition* (sleeping vs. watching video) were implemented in separate experimental sessions at least 1 week apart to minimize practice effects. In the sleep condition, the room was completely shaded and the participants had the opportunity to sleep for 90 min. In the video condition, the participants watched two documentary videos about Scandinavia on the LCD screen of a laptop computer while the room was illuminated by office neon tubes.

**FIGURE 2:**
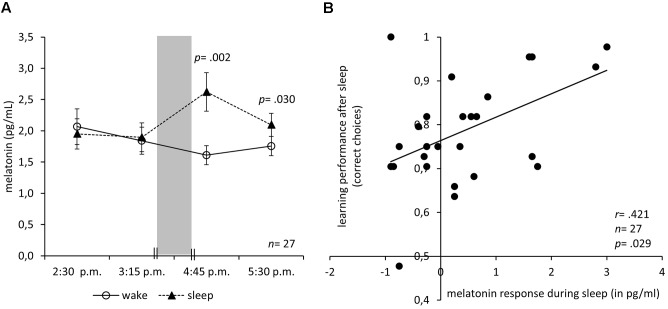
Melatonin response during nap correlates with subsequent learning. **(A)** Mean and SEM of saliva melatonin levels before and after an interval either with sleep in darkness (nap condition) or wake in bright light (video condition). The 90 min sleep/wake interval is indicated by the gray bar. **(B)** Correlation of the melatonin response with the learning performance after the sleep interval in darkness (nap condition) with a regression line. Note that the guessing frequency is 0.5 correct choices.

The participants were instructed to sleep from 11:30 p.m. to 7 a.m. the night before each experimental session. They arrived at 2:00 p.m. in the lab, received instructions, and, in the sleep condition, the electrodes were affixed. Thereafter, the participants collected the first saliva sample (2:30 p.m.), worked on the first learning task, collected the second saliva sample (3:15 p.m.), and rated their mood (see also **Figure 2A**). After the interval (3:15–4:45 p.m.) encompassing the manipulation, the participants again rated their mood, collected the third saliva sample (4:45 p.m.), worked on the second learning task, and collected a final saliva sample (5:30 p.m.). The order of the parallel versions of the learning task and the order of the *Conditions* were approximately counterbalanced.

### Data Analysis

To evaluate the effect of the manipulation on learning performance, saliva melatonin levels, or mood, we computed ANOVAs with the between-subject factor *Condition* (video vs. nap) and the within-subject factor *Time* (before vs. after the manipulation). In the case of significant effects, *post hoc* Bonferroni-adjusted *t*-contrasts were computed. To obtain a robust measure for the melatonin response, we used the mean melatonin levels before the intervals as a baseline and computed the difference between the first post measurement and the baseline. Positive values indicated an increase in melatonin levels during the interval. Note that we abstained from log-transforming the melatonin values because the values did not spread across orders of magnitude as in studies assessing the dim-light melatonin onset. In fact, the range was only 6.7 pg/mL. In an exploratory analysis, we defined increases of at least 0.6 pg/mL as a response and compared the frequencies in the nap versus wake conditions using a McNemar’s test. To evaluate possible correlations of sleep, arousal, learning performance, and melatonin, we used Pearson correlation coefficients. To exclude the possibility that sleep or arousal was confounded with melatonin regarding the correlation with learning performance, we calculated regression analyses using a bootstrap simulation with 5000 samples to obtain robust estimates of the significance of the regression coefficients.

## Results

The *manipulation check* revealed that the participants slept 68.0 min on average in the nap condition. However, there was considerable variation in the length of the total sleep time (range: 7.5–91.0 min, *SD* = 21.6 min), the amounts of REM-sleep (range: 0–16.0 min, *SD* = 5.2 min), the amount of light sleep (S1+S2; range: 7.5–75.5 min, *SD* = 15.9 min), and slow-wave sleep (S3+S4; range: 0–50.5 min, *SD* = 13.3 min, further details are reported in **Table 1**). Sleep did not affect arousal and valence of mood differently than the control condition. An ANOVA of the arousal ratings with the between-subject factor *Condition* and the within-subject factor *Time* (before vs. after the manipulation) revealed a main effect of *Time* (*F*_1,26_ = 10.70; *p* = 0.003) but no main effect of *Condition* (*F*_1,26_ = 0.09; *p* = 0.771) and no interaction of *Condition* and *Time* (*F*_1,26_ = 0.12; *p* = 0.729). *Post hoc* Bonferroni-adjusted *t*-contrasts confirmed that the participants’ arousal dropped while watching the video (*p* = 0.009) as well as during the nap (*p* = 0.036). The valence ratings, on the other hand, were not influenced by the manipulation. An ANOVA of the valence ratings revealed no main effects of *Time* (*F*_1,26_ = 1.34; *p* = 0.257), no main effect of *Condition* (*F*_1,26_ = 0.89; *p* = 0.355), and no interaction of *Condition* and *Time* (*F*_1,26_ = 0.14; *p* = 0.713). Furthermore, the total sleep time negatively correlated with the arousal ratings before the nap (*r* = -0.505, *n* = 27, *p* = 0.007) but not after the nap (*r* = -0.121, *n* = 27, *p* = 0.546), indicating that participants with low arousal (i.e., wakefulness) slept longer than participants with high arousal.

**FIGURE 3:**
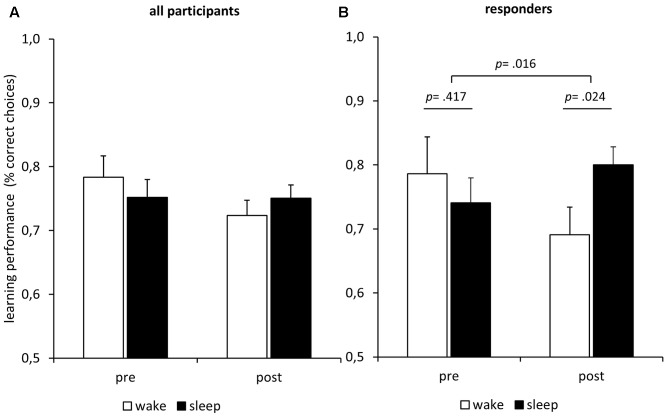
Influence of sleep/darkness on feedback learning. **(A)** Learning performance (mean ± SEM) improved during the interval irrespective of condition. Note that the guessing frequency is 0.5 correct choices. **(B)** Only in the melatonin responders (*n* = 11) did learning performance increase more during sleep in darkness than during wake in bright light.

Does sleep improve *post-sleep reward learning*? The results do not confirm this hypothesis (**Figure 3A**). An ANOVA of the learning performance showed no main effect of *Condition* (*F*_1,26_ = 0.01; *p* = 0.925), no main effect of *Time* (*F*_1,26_ = 1.63; *p* = 0.214), and no interaction of *Condition* and *Time* (*F*_1,26_ = 1.69; *p* = 0.206). Although there was considerable variation in sleep duration and the amount of sleep in different stages, we did not find any correlation of performance after the nap with total sleep time (*p* = 0.485), light-sleep (*p* = 0.511), slow-wave sleep (*p* = 0.766), or any single sleep stage (REM-sleep, S1, S2, S3, S4; all *p* > 0.100). In summary, there is no indication of any sleep-specific improvement or practice effect. To check whether any sleep parameter would predict learning performance after sleep we calculated two regression analyses. In the first regression, we entered the amounts of REM-sleep, S1, S2, S3, and S4 as predictors and the learning performance after sleep as criterion. The analysis revealed no significant regression equation (*F*_5,21_ = 0.80; *p* = 0.561) and no sleep stage predicted learning performance (all *p* > 0.180). As it has been hypothesized that especially REM sleep might foster subsequent encoding of emotional memories (Kaida et al., 2015), we computed an additional regression analysis to check whether any REM-sleep parameter would predict learning performance. We entered REM density, REM latency, and REM duration as predictors and the learning performance after sleep as criterion. The analysis revealed no significant regression equation (*F*_3,12_ = 1.14; *p* = 0.372) and no parameter predicted learning performance (all *p* > 0.202).

Is it possible to elicit an increase of *melatonin* in the afternoon, long before a circadian dim-light melatonin onset is to be expected? **Figure 2A** depicts the melatonin levels at 2:30 p.m. and 3:15 p.m. before the manipulation as well as 4:45 p.m. and 5:30 p.m. after the manipulation. The interval containing the manipulation is depicted as a gray bar. In fact, sleeping in complete darkness increased the melatonin levels. An ANOVA of the saliva melatonin levels with the between-subject factor *Condition* and the within-subject factor *Time* (before: 2:15 p.m. and 2:45 p.m., after: 4:15 p.m. and 4:45 p.m.) revealed a significant interaction of *Condition* and *Time* (*F*_3,78_ = 7.44; *p* < 0.001) but no significant main effects of *Condition* (*F*_1,26_ = 4.04; *p* = 0.055) or *Time* (*F*_3,78_ = 0.99; *p* = 0.402). However, there was a slight trend toward higher melatonin levels in the sleep condition. *Post hoc* Bonferroni-adjusted *t*-contrasts showed that before the manipulation melatonin levels did not differ between conditions (*p* = 0.606 at 2:15 p.m.; *p* = 0.799 at 2:45 p.m.). After the manipulation, melatonin levels were higher under the sleep condition (*p* = 0.002 at 4:15 p.m.; *p* = 0.030 at 4:45 p.m.).

Moreover, melatonin might have facilitated learning after the sleep (**Figure 2B**): The height of the melatonin response in the nap condition positively correlated with the learning performance after the sleep (*r* = 0.421, *n* = 27, *p* = 0.029) but not with learning performance before (*r* = 0.038, *n* = 27, *p* = 0.852). The correlation of the melatonin response with the learning performance after the sleep remained significant when controlling for melatonin before sleep (partial *r* = 0.389, *df* = 24, *p* = 0.050) or learning before sleep (partial *r* = 0.423, *df* = 24, *p* = 0.031). Moreover, the correlation of melatonin after sleep and learning after sleep was significantly higher than that of melatonin before sleep and learning after sleep (*z* = 2.161, *p* = 0.015). In the wake condition, the amplitude of the melatonin response did not correlate with the learning performance before (*r* = -0.032, *n* = 27, *p* = 0.874) nor after (*r* = 0.123, *n* = 27, *p* = 0.542) the wake interval. To exclude the possibility that subjective arousal or valence caused the increase in learning performance, we calculated a regression analysis with arousal and valence ratings as predictors and learning performance after the manipulation as a criterion. The analysis revealed no significant regression equation (*F*_2,24_ = 1.97; *p* = 0.161) and neither arousal (*p* = 0.433) nor valence (*p* = 0.278) predicted learning performance.

**FIGURE 4:**
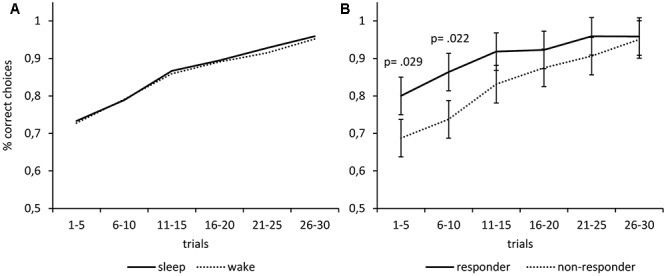
Melatonin but not sleep fosters subsequent feedback learning. **(A)** The learning performance was almost identical after waking vs. sleep. **(B)** Participants who showed an increase in saliva melatonin during the nap (responders) subsequently displayed faster feedback learning as compared to non-responders. Differences occur very early in the task (trials 1–5) indicating that working memory might have supported feedback learning. The *p*-values correspond to two-tailed *t*-tests.

In an exploratory analysis, we classified participants as responders if their melatonin-response reached at least a magnitude twice as large as the analytic sensitivity of 0.3 pg/mL. We calculated the melatonin response as the mean melatonin level after the manipulation minus the mean melatonin level before the manipulation. An exact McNemar’s test using the binomial distribution confirmed that there were significantly (*p* = 0.002) more responders under the nap condition (*n* = 11; 40.7%) than under the wake condition (*n* = 2; 7.4%). We repeated the ANOVA of the learning performance reported above using only the data of the 11 responders (**Figure 3B**). Again, we found no main effects of *Time* (*F*_1,10_ = 0.28; *p* = 0.607) and no main effect of *Condition* (*F*_1,10_ = 0.64; *p* = 0.442). However, there was a significant interaction of *Time* and *Condition* (*F*_1,10_ = 8.43; *p* = 0.016), indicating that the melatonin-responders’ learning performance improved more during the sleep interval than during the wake interval. *Post hoc* Bonferroni-adjusted *t*-contrasts confirmed that the melatonin-responders showed better learning performance after the sleep interval as compared to the wake interval (*p* = 0.024), while there were no differences before the respective intervals (*p* = 0.417). The same analysis using the data of the non-responders revealed no such interaction effect of *Time* and *Condition* on learning performance (*F*_1,15_ = 0.01; *p* = 0.909), no main effect of *Condition* (*F*_1,15_ = 0.95; *p* = 0.344), and no main effect of Time (*F*_1,15_ = 1.35; *p* = 0.263). Furthermore, we computed separate learning curves for responders and non-responders (**Figure 4B**). The responders not only showed an increase in saliva melatonin during the nap but also displayed faster feedback learning after the nap. Significant differences occurred very early in the task (trials 1–5, *t*_25_ = 2.32, *p* = 0.029) indicating that working memory might have supported feedback learning. For comparison, **Figure 4** also shows learning curves after the sleep versus the wake condition (**Figure 4A**). The curves show only minor differences in percentages of correct responses differing in the order of 1%.

Finally, we checked whether gender, contraceptives, or smoking sporadically had any influence on the melatonin levels before or after each condition using *t*-tests. Melatonin levels did not differ between genders (all *p* > 0.248), between women taking contraceptives or not (all *p* > 0.196), nor between participants smoking sporadically or not (all *p* > 0.369).

## Discussion

In our nap-study, *sleep did not improve reward learning* as compared to a wake condition. Despite considerable variation of the amount of sleep in different stages, no sleep parameter correlated with post-sleep learning performance. Furthermore, neither the sleep stage durations nor REM parameters predicted post-sleep learning performance. Recall, that the synaptic homeostasis hypothesis states that sleep renormalizes synaptic weights, thereby refreshing the ability to learn night by night (Tononi and Cirelli, 2014). The hypothesis stood to the test whenever the influence of sleep on declarative or hippocampus-dependent learning was tried (for an overview see Tononi and Cirelli, 2014; Cirelli and Tononi, 2015). However, several studies did not find a beneficial effect of sleep on post-sleep procedural learning. For example, three studies found improved declarative learning after sleep but no improvement in a serial reaction time task which assesses implicit motor sequence learning and is deemed to be relatively independent of the hippocampus (Mander et al., 2011; Van Der Werf et al., 2011; Antonenko et al., 2013). Instead, implicit motor sequence learning depends on the basal ganglia and the dopaminergic system (Hardwick et al., 2013; Hayes et al., 2015). Regarding the neuronal underpinnings, reward learning is very similar to motor sequence learning. Reward learning is also relatively independent of the hippocampus and depends on the striatum and the dopaminergic system (Schultz, 2015). This might explain why in our study sleep did not foster post-sleep reward learning. It seems that mostly hippocampus-dependent forms of learning require renormalization of synaptic weights during sleep and that striatum-dependent learning uses different mechanisms to renew and protect learning ability.

An interesting finding of our study is that *a daytime nap in darkness increases the saliva melatonin* level whereas as watching a video in daylight does not. Note that the melatonin levels were also higher half an hour after sleep and despite proper rinsing before sample collection. Therefore, it is highly unlikely that the melatonin values could have been inflated due to less swallowing during sleep. To our knowledge, this is the first study showing a melatonin response during the day, long before the circadian dim-light melatonin onset is to be expected. In older articles from the ‘90s, it was common to display individual melatonin curves of multiple subjects. A thorough review of these studies revealed that in some subjects a small melatonin increase can be observed in the afternoon at around 4:00 p.m. (Laakso et al., 1990; Hashimoto et al., 1997; Duffy et al., 1999). Although the circumstances of these increases are unknown, the older studies support the notion that small melatonin responses can already occur during the day in some individuals. In another study from the 70s, two healthy men were observed for six consecutive days under standardized, light-dark conditions, and with afternoon naps on 3 days (Vaughan et al., 1979). Near the middle of the light period, occasional melatonin peaks occurred but they were not consistently related to the afternoon naps. The authors concluded that melatonin secretion followed an episodic pattern. This claim is supported by a study of five young, healthy men who also displayed episodic melatonin secretion patterns during the day (Weinberg et al., 1979). In our study, about 41% of the participants showed a substantial melatonin response during daytime and only in the nap condition. This might explain why this phenomenon has not been reported in earlier studies. Future studies should try to identify factors that distinguish daytime responders from non-responders. However, the systematic increase of daytime melatonin in our study offers the possibility to study the effects of physiological melatonin secretion on neuropsychological functions like learning.

Another new finding of our study is the *correlation of the melatonin response and reward learning* after the sleep/darkness interval: the greater the melatonin response was, the better the participants learned. This suggests that melatonin might have improved reward learning. The correlation between melatonin response and learning was only found in the nap condition and only after the nap. Therefore, it seems unlikely that a trait factor produced the correlation. Moreover, learning performance only increased in the responders during the sleep interval but not during the wake interval. Another alternative explanation would be that sleep or mood might have caused better post-sleep learning. However, neither subjective arousal, nor valence, nor any sleep parameter predicted post-sleep reward learning. Other nuisance variables might be seen in gender, contraceptives, or sporadic smoking. Yet, the within-subject design ensures that these variables would have influenced both conditions the same way. Also, there were no significant effects of gender, contraceptives, or sporadic smoking on saliva melatonin levels in either condition. In summary, the conclusion that the melatonin response facilitated reward learning seems valid.

Our finding that the melatonin response in the nap condition correlated with reward learning matches the study by Rimmele et al. (2009). The authors reported that externally administered melatonin during daytime increased learning under stress in a declarative memory task. Although this study supports our findings on the behavioral level, the mechanisms by which melatonin can increase learning are still unknown (Zakaria et al., 2016). Moreover, melatonin may act differently concerning hippocampus-dependent versus striatum-dependent learning. On the one hand, melatonin agonists are suspected to inhibit long-term potentiation via MT2-receptors in the hippocampus (Larson et al., 2006; Liu et al., 2016). On the other hand, melatonin given as a medication has been shown to modulate and protect the nigrostriatal dopaminergic system from oxidative stress in Parkinson’s disease (Zisapel, 2001; Carriere et al., 2016). Moreover, MT1 and MT2-receptors are expressed in the striatum and are supposed to regulate reward-related behaviors according to the circadian rhythm in rodents (Clough et al., 2016). Therefore, it seems possible that endogenously secreted melatonin may act on the dopaminergic reward system and improve reward learning. However, whether melatonin improves or impairs learning seems to depend on many factors: Melatonin effects depend on whether an animal is diurnally vs. nocturnally active, on the time of administration in the rest-activity cycle, and on externally administered vs. endogenously secreted (Gorfine and Zisapel, 2007). Furthermore, high doses of externally administered melatonin may be counterproductive regarding effects on memory and learning (Foster et al., 2014). Therefore, our pilot study can only be a first step toward the investigation of the effects of endogenously secreted melatonin on reward learning.

An alternative explanation might be that melatonin fostered working memory rather than reward learning. As Collins et al. (2014) pointed out, most reward learning paradigms also engage higher order cognitive processes which are supported by the prefrontal cortex (Collins and Frank, 2012). The authors devised an instrumental learning task that allowed disentangling reinforcement learning from working memory by providing feedback (“reward”) as well as varying working-memory load. A combined model consisting of a reinforcement-learning model and a working-memory model explained data from a sample genotyped for polymorphisms affecting the prefrontal cortex or the basal ganglia better than separate models (Collins and Frank, 2012). The combined model also allowed showing that apparent reinforcement learning deficits in patients suffering from schizophrenia can be explained entirely by working memory deficits instead of reward learning deficits (Collins et al., 2014, 2017). In our study, the participants worked on very simple probabilistic reinforcement learning tasks with only two stimuli at a time. This makes it easy to track feedback in working memory. Moreover, differences between melatonin-responders and non-responders occurred very early during learning (trials 1–5). Reward learning, in general, is a rather slow process as compared to working memory (Collins and Frank, 2012). Therefore, it seems possible that in our study melatonin fostered working memory rather than reward learning. However, further studies are needed to disentangle the components of learning which are possibly fostered by melatonin. Moreover, future studies should include an additional control condition to further rule out the interpretation that simply rest (in darkness) would be sufficient to improve subsequent feedback learning. However, the correlation of melatonin and subsequent learning in our study suggests that the effect is specific for melatonin. Otherwise, all subjects would have shown improved learning following rest/sleep.

A limitation of our study is that it is not entirely clear whether the melatonin response was elicited by the darkness or the sleep during the nap period. Previous research strongly suggests that darkness was the crucial factor (Pandi-Perumal et al., 2007). While the nap condition took place in complete darkness, the wake condition took place during daylight with additional office lighting and in front of a monitor with LCD-background light. Note that the high amount of light in the blue spectrum from the LCD monitor and the office lighting is supposed to suppress melatonin secretion as opposed to darkness which is supposed to disinhibit melatonin secretion (Cajochen et al., 2011). It seems unlikely that sleep – not darkness – triggered the melatonin response because the melatonin response in the nap condition did not correlate with the previous amount of sleep. However, we suggest further experiments evaluating the effect of a sleep condition, a wake condition in darkness, and a wake condition in bright light on melatonin during the daytime.

In summary, we found that a nap in complete darkness during daytime can already elicit a melatonin response and that the magnitude of this response positively correlates with subsequent reward learning. The difference between melatonin-responders and non-responders occurred very early during learning indicating that melatonin might have improved working memory rather than reward learning. Either way melatonin might be a useful agent to improve learning in clinical samples. Moreover, our results underline the importance of a healthy circadian melatonin secretion, especially regarding the exposure to bright light during the evening, which inhibits the physiological melatonin secretion. Future studies should investigate this effect of endogenous melatonin on learning at different points in time in the circadian rhythm using different amounts of light to block or disinhibit melatonin secretion. Moreover, future studies should use paradigms disentangling working memory and reward learning to clarify which aspect of human feedback learning might profit from melatonin.

## Ethics Statement

This study was carried out in accordance with the recommendations of Declaration of Helsinki. All participants gave written informed consent. The study protocol was approved by the ethics committee of the medical faculty of the University of Kiel.

## Author Contributions

CW designed the study, wrote the manuscript, and programmed the software. CW and VD collected the data. CW, VD, DS, AP-K, and LB analyzed and interpreted the data, approved the manuscript, and agreed to be accountable for all aspects of the work. VD, AP-K, and LB revised the manuscript.

## Conflict of Interest Statement

The authors declare that the research was conducted in the absence of any commercial or financial relationships that could be construed as a potential conflict of interest.
